# Chemogenetic Suppression of Medial Prefrontal-Dorsal Hippocampal Interactions Prevents Estrogenic Enhancement of Memory Consolidation in Female Mice

**DOI:** 10.1523/ENEURO.0451-18.2019

**Published:** 2019-04-16

**Authors:** Jennifer J. Tuscher, Lisa R. Taxier, Jayson C. Schalk, Jacqueline M. Haertel, Karyn M. Frick

**Affiliations:** Department of Psychology, University of Wisconsin-Milwaukee, Milwaukee, WI 53211

**Keywords:** DREADD, estradiol, hippocampus, mouse, prefrontal cortex, spine density

## Abstract

The importance of the dorsal hippocampus (DH) in mediating the memory-enhancing effects of the sex-steroid hormone 17β-estradiol (E_2_) is well established. However, estrogen receptors (ERs) are highly expressed in other brain regions that support memory formation, including the medial prefrontal cortex (mPFC). The mPFC and DH interact to mediate the formation of several types of memory, and behavioral tasks that recruit the mPFC are enhanced by systemic E_2_ administration, making this region a prime candidate for investigating circuit-level questions regarding the estrogenic regulation of memory. Further, infusion of E_2_ directly into the DH increases dendritic spine density in both the DH and mPFC, and this effect depends upon rapid activation of cell-signaling pathways in the DH, demonstrating a previously unexplored interaction between the DH and mPFC that led us to question the role of the mPFC in object memory consolidation and the necessity of DH-mPFC interactions in the memory-enhancing effects of E_2_. Here, we found that infusion of E_2_ directly into the mPFC of ovariectomized mice increased mPFC apical spine density and facilitated object recognition and spatial memory consolidation, demonstrating that E_2_ in the mPFC increases spinogenesis and enhances on memory consolidation. Next, chemogenetic suppression of the mPFC blocked the beneficial effects of DH-infused E_2_ on memory consolidation, indicating that systems-level DH-mPFC interactions are necessary for the memory-enhancing effects of E_2_. Together, these studies provide evidence that E_2_ in the mPFC mediates memory formation, and reveal that the DH and mPFC act in concert to support the memory-enhancing effects of E_2_ in female mice.

## Visual Abstract

**Figure:**
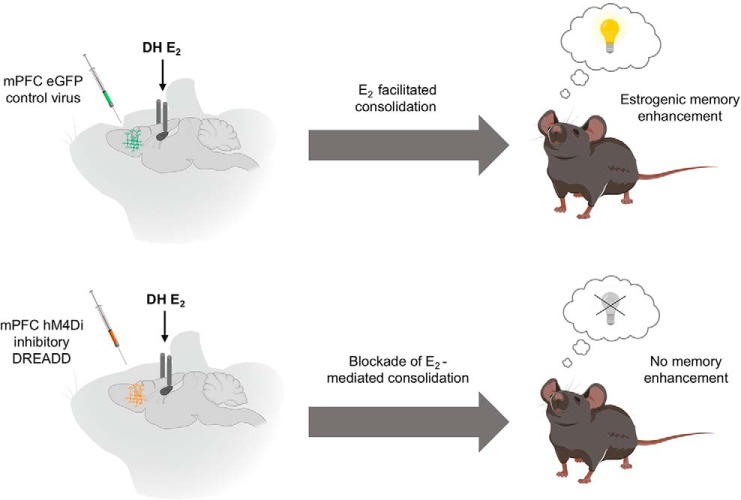

## Significance Statement

Estrogens influence the prevalence and severity of certain psychiatric and neurodegenerative disorders, many of which are characterized by impaired medial prefrontal cortex (mPFC) and dorsal hippocampus (DH) function. Myriad findings demonstrate that 17β-estradiol (E_2_) regulates hippocampal plasticity and memory, however, far less is known about estrogenic regulation of other interconnected brain regions, like the mPFC. Here, we report that mPFC E_2_ infusion in ovariectomized mice increases mPFC spine density and facilitates memory consolidation, and that chemogenetic inactivation of the mPFC blocks the memory-enhancing effects of DH-infused E_2_. These findings suggest an essential role for the mPFC, alone and in concert with the DH, in mediating the memory-enhancing effects of E_2_. Such circuit-level interactions may be critical to understanding how E_2_ regulates memory.

## Introduction

Sex-steroid hormones have a broad impact on the neural circuitry that supports learning and memory, yet much remains unknown about the cellular and circuit-level mechanisms through which they exert their effects. The potent estrogen 17β-estradiol (E_2_) can regulate neuronal excitability and spinogenesis in the dorsal hippocampus (DH) and medial prefrontal cortex (mPFC), brain regions important for cognitive function that are compromised during aging and in numerous neuropsychiatric disorders (Godsil et al., 2013; Sampath et al., 2017). Memory consolidation, a process which requires coordinated effort between the hippocampus and mPFC, is facilitated by systemic injection or direct infusion of E_2_ into the DH of female rodents (Tuscher et al., 2015). However, the specific mechanisms through which E_2_ enhances memory consolidation remain poorly understood, and little is known about how interactions between the DH and mPFC might contribute to estrogenic regulation of memory.

The object recognition (OR) and object placement (OP) tasks involve the integration of “what” and “where” components of memory, and are commonly used to assess episodic-like memory in rodents (Dere et al., 2005; Barker et al., 2017; Eichenbaum, 2017). Previous work has demonstrated that direct DH infusion of E_2_ immediately after object training can extend the delay at which ovariectomized mice can recall training objects or locations, and that this enhanced memory consolidation depends on E_2_-mediated activation of the extracellular signal-regulated kinase (ERK) and mammalian target of rapamycin (mTOR) cell-signaling pathways in the DH (Fernandez et al., 2008; Fortress et al., 2013). Recent research has also shown that E_2_-induced increases in spinogenesis in both the DH and mPFC rely on activation of ERK and mTOR signaling in the DH (Tuscher et al., 2016a), highlighting putative systems-level interactions between these brain regions that may be important for memory formation in female rodents. However, the extent to which DH-mPFC interactions are necessary for the memory enhancing effects of E_2_ remains unknown. Moreover, the mPFC expresses all major estrogen receptor (ER) subtypes (Almey et al., 2014), suggesting E_2_ may act directly in the mPFC to impact spinogenesis or enhance memory consolidation. The idea that E_2_ may act within the mPFC to improve neuroplasticity and memory is further supported by studies showing systemic E_2_ administration can increase mPFC spine density, alter glutamatergic synaptic transmission, and improve memory in tasks that involve the mPFC, including the radial arm maze, Y maze, OR, and OP (Luine et al., 1998; Luine, 2016; Velazquez-Zamora et al., 2012; Galvin and Ninan, 2014).

Therefore, the present study sought to determine the extent to which E_2_ can act directly in the mPFC to regulate object recognition and spatial memory consolidation, and the necessity of DH-mPFC interactions for the memory-enhancing effects of DH E_2_ infusion. We first delivered E_2_ directly to the mPFC of ovariectomized mice immediately after object training to assess the effects of E_2_ on episodic memory consolidation and spine density in the mPFC and DH. mPFC-infused E_2_ increased mPFC apical spine density and enhanced object recognition and spatial memory consolidation, indicating that E_2_ can act directly in the mPFC to regulate both dendritic morphology and memory formation. Next, we used designer receptors exclusively activated by designer drugs (DREADDs) to inactivate the mPFC immediately before DH-E_2_ infusion to determine whether coordination between the DH and mPFC is necessary for DH-infused E_2_ to enhance memory consolidation in ovariectomized mice. Chemogenetic suppression of the mPFC prevented DH-infused E_2_ from enhancing both object recognition and spatial memory consolidation, suggesting that coordinated activity in the DH and mPFC is necessary for E_2_ to facilitate memory formation. These data provide evidence for a key role of the mPFC, and of DH-mPFC interactions, in the memory-enhancing effects of E_2_ in ovariectomized mice.

## Materials and Methods

### Subjects

Female C57BL/6 mice were obtained from Taconic Biosciences at 9–12 weeks of age and were housed individually in a room with a 12/12 h light/dark cycle and *ad libitum* access to food and water. Experimenters conducting behavioral testing were blind to treatment status. All procedures were performed in accordance with the University of Wisconsin-Milwaukee Institutional Animal Care and Use Committee’s regulations and are consistent with National Institutes of Health Guidelines for the Care and Use of Laboratory Animals.

### Surgery

Mice were anesthetized with isoflurane (5% for induction, 2% for maintenance) in 100% oxygen and then bilaterally ovariectomized and implanted with bilateral cannulae into the mPFC or DH as described previously (Kim et al., 2016; Tuscher et al., 2016b). DH-cannulated mice also received bilateral mPFC injections of DREADD virus as described below. Ovariectomy, cannulae implantation, and virus injections occurred during the same surgical session. For analgesia, mice received carprofen MediGel 1 d before surgery and subcutaneous injection of 5-mg/kg Rimadyl immediately after surgery.

#### mPFC cannulation

Immediately after ovariectomy, mice were implanted with stainless steel bilateral guide cannulae (Plastics One) aimed at the mPFC (*n* = 12–13/group; 1.8 mm AP, ±0.3 mm ML, –2.3 mm DV) and fixed to the skull with dental cement (Darby Dental Supply). Dummy cannulae were inserted into guide cannulae to prevent clogging. Mice recovered one week before behavioral testing.

#### mPFC DREADD delivery and DH cannulation

Immediately after ovariectomy, bilateral injections were made into the mPFC (*n* = 9–11/group; 1.9 mm AP, ±0.3 mm ML, –2.8 mm DV) using a 10 µl Hamilton syringe, which was first lowered to –2.8 mm ventral to the skull surface and held in place for 2 min to create a pocket for the first virus injection, as described previously (Tuscher et al., 2018). The syringe was then raised 0.1 mm, and hM4Di DREADD virus (AAV-CamKIIα-HA-hM4Di-IRES-mCitrine, 2.1 × 10^12^ particles/ml, serotype 8, UNC Vector Core), eGFP control virus (AAV-CamKIIα-eGFP, 2.1 × 10^12^ particles/ml, serotype 8, UNC Vector Core), or saline (sham condition) was delivered using a syringe pump (KD Scientific) at a rate of 0.2 µl/min for 2 min, for a total of 0.4 µl/infusion. The syringe was then raised 0.2 mm, and a second infusion of the same volume was delivered at the same rate for a total of 0.8 µl/hemisphere. The syringe remained in place for 8 min for after each injection to allow for virus diffusion, and was then slowly retracted. Mice were then implanted with stainless steel bilateral guide cannulae aimed at the DH (–1.7 mm AP, ±1.5 mm ML, –2.3 mm DV) as described previously (Tuscher et al., 2016a). Mice received presurgical and postsurgical analgesia as described above and were given three weeks for recovery and to allow sufficient time for viral expression before behavioral testing.

### Drugs and infusions

Infusions into the mPFC or DH were conducted as described previously (Kim et al., 2016; Tuscher et al., 2016b). Briefly, cyclodextrin-encapsulated 17β-E_2_ (Sigma-Aldrich) was dissolved in sterile 0.9% saline to a concentration of 10 µg/µl, and infused bilaterally into the DH or mPFC immediately after training. The vehicle, 2-hydroxypropyl-β-cyclodextrin (HBC; Sigma-Aldrich), was dissolved in saline to the same concentration of cyclodextrin present in the cyclodextrin-encapsulated E_2_ solution. Infusions were conducted at a rate of 0.5 μl/min for 1 min per hemisphere as described previously (Fernandez et al., 2008; Fortress et al., 2013), resulting in a dose of 5-µg E_2_/hemisphere.

For DREADD experiments, stock solutions of clozapine-N-oxide (CNO, Cayman Chemical) were prepared by dissolving CNO in 100% dimethyl sulfoxide (DMSO) at a concentration of 100 mg/ml, and storing 10-µl aliquots at –20°C, as described previously (Tuscher et al., 2018). On the day of injection, CNO stock was thawed and diluted to 1 mg/ml in a solution of sterile 0.9% saline containing 2% DMSO. Intraperitoneal injections of 1-mg/kg CNO were administered immediately after training, followed directly by bilateral DH infusion of vehicle or E_2_.

### Behavioral testing

OR and OP protocols used to measure object recognition and spatial memory were conducted as described previously (Boulware et al., 2013; Fortress et al., 2013; Kim et al., 2016). Memory consolidation in both tasks is enhanced by E_2_ and involves the DH (Tuscher et al., 2015). One week after mPFC cannula surgery or three weeks after DREADD/DH cannula surgery, mice were handled for 1 min/d for 3 d before habituation. Mice were then habituated for two consecutive days by allowing them to explore the empty white arena (60 × 60 × 47 cm) for 5 min/d. During training, mice accumulated 30 s exploring two identical objects placed in the upper left and right corners of the arena. Time spent with the objects was recorded using ANY-maze tracking software (ANY-maze, RRID:SCR_014289). Immediately after training, mice were injected or infused as described above and then returned to their home cage. Post-training injections were used to pinpoint treatment effects to the memory consolidation period while minimizing potential confounding effects on performance factors (e.g., motivation, anxiety) on the measurement of memory consolidation (McGaugh, 1989; Frick and Gresack, 2003). OR memory was tested 48 h later; intact OR memory was demonstrated if the mice spent more time than chance (15 s) with the novel object during testing. OP training and testing were identical to OR, except that testing was conducted 24 h after training, and involved moving one of the identical training objects to a new location during testing. The 48-h and 24-h delays between training and testing in OR and OP, respectively, were used because mice infused with E_2_ into the DH demonstrate enhanced OR and OP memory consolidation at these time points (Tuscher et al., 2016b; Tuscher et al., 2018). All mice were trained and tested in both tasks with the order of testing counterbalanced such that half of the mice completed OR first and half completed OP first.

### Histologic verification of DREADD expression

Three weeks after eGFP or DREADD virus surgeries, a subset of mice (*n* = 3) were anesthetized with isoflurane and perfused with 4% paraformaldehyde (PFA) in 1× PBS to confirm virus expression at the onset of behavioral training. Whole mouse brains were removed and postfixed in 1× PBS/4% PFA overnight, followed by dehydration in a 1× PBS/30% sucrose solution. Tissue was sectioned on a cryostat (40 µm) and free-floated in 1× PBS until it was mounted onto microscope slides (VWR) using aqueous mounting medium containing the nuclear stain DAPI (Santa Cruz). Fluorescent images were captured using an Olympus Fluoview FV1200 confocal microscope and accompanying software.

### Golgi staining and spine counting

Two weeks after completion of behavioral testing, mice received mPFC infusions of E_2_ or vehicle and were killed 2 h later to assess E_2_-mediated spine density changes in the DH and mPFC. This time point was selected because DH infusion of E_2_ significantly increases dendritic spine density in both brain regions 2 h later (Tuscher et al., 2016a). Whole brains were collected and Golgi impregnation was performed as described previously (Frankfurt et al., 2011) using the Rapid GolgiStain kit (FD NeuroTechnologies). Briefly, secondary basal dendrites and tertiary apical dendrites were counted blindly from pyramidal neurons in CA1 and Layer II/III of the prelimbic region of the mPFC. Dendrites from sex cells/region/brain were included in the analysis, and six to eight brains were quantified/group. Neurons were chosen for analysis if their cell bodies and dendrites were well impregnated, and dendrites were continuous and clearly distinguishable from adjacent cells. Spines were counted on an Olympus BX51WI microscope under oil (100×) using Neurolucida version 11.08 (MBF Bioscience; RRID:SCR_001775). Spine density was calculated by dividing spine number by dendrite length, and data expressed as number of spines/10-µm dendrite.

### Experimental design and statistical analysis

Power analyses indicate that at least nine mice per group per behavioral experiment and nine mice/group per spine counting experiment will provide 90% power to detect small effect sizes at *p* = 0.05, two-tailed. In the first experiment, ovariectomized mice were trained in OR and OP, and then immediately received bilateral mPFC infusion of vehicle or 5 µg/hemisphere E_2_ (*n* = 10–13/group) to determine the extent to which E_2_ in the mPFC could regulate memory consolidation. Memory was tested 24 h (OP) or 48 h (OR) later. Two weeks after the conclusion of behavioral testing, mice received mPFC infusions of vehicle or E_2_, and whole brains were collected 2 h later for Golgi staining and dendritic spine density analyses (*n* = 6–8/group). In the second experiment, a new set of ovariectomized mice were cannulated in the DH and injected with saline (sham) or an AAV8 viral vector containing eGFP control virus or the inhibitory hM4Di DREADD in the mPFC. A subset of mice (*n* = 3/group) injected with eGFP or hM4Di were perfused three weeks later to confirm virus expression at the initiation of behavioral testing. Immediately after behavioral training, mice were injected with 1-mg/kg CNO and then received DH infusion of vehicle or 5 µg/hemisphere E_2_. Memory was tested 24 h (OP) or 48 h (OR) later.

Statistical analyses were conducted using GraphPad Prism 6 software (RRID:SCR_002798). To determine whether learning occurred within each group, behavioral data were analyzed using one sample *t* tests to assess whether the time each group spent exploring the novel (OR) or moved (OP) objects differed from chance (15 s; Tuscher et al., 2016b, 2018). This analysis was used because time spent with the objects is not independent; time spent with one object reduces time spent with the other (Frick and Gresack, 2003). Between-group comparisons were assessed with Student’s *t* tests or one-way ANOVA. Effects of mPFC vehicle and E_2_ infusion were compared using Student’s *t* tests. For other experiments involving more than two treatment groups, between-group differences were measured using one-way ANOVAs with treatment as the independent variable, followed by Fisher’s LSD *post hoc* tests when appropriate (Tuscher et al., 2016b, 2018). For spine density analyses, Student’s *t* tests were used to determine the effect of E_2_ treatment on spine density in each brain region, followed by *post hoc* tests when appropriate. Statistical significance was determined as *p* ≤ 0.05.

## Results

### mPFC E_2_ infusion immediately after training enhances memory consolidation

**Figure 1.**
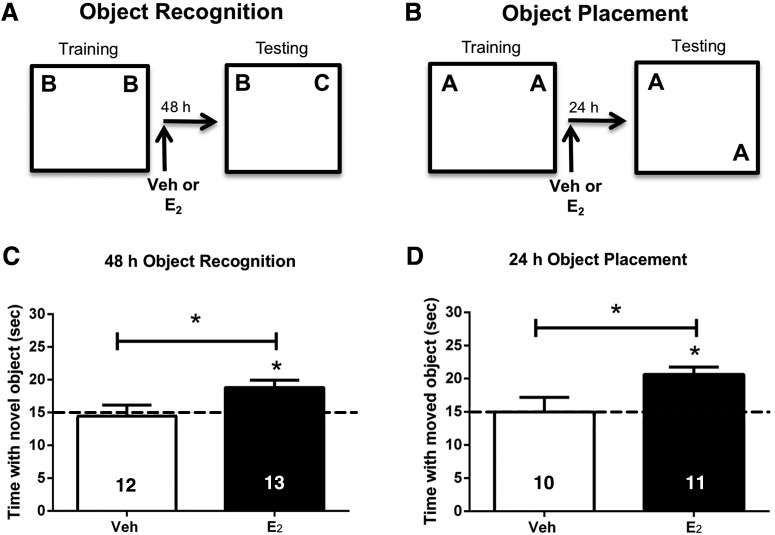
Infusion of E_2_ into the mPFC immediately after training enhances memory consolidation. Mice infused with E_2_ directly into the mPFC spent significantly more time than chance (dashed line at 15 s) with the novel object (***A***, ***C***) when tested 48 h after training, or with the moved object (***B***, ***D***) 24 h after training. Mice infused with vehicle (Veh) into the mPFC did not spend more time than chance with the novel or moved objects. These data suggest that E_2_ can improve the consolidation of object memories by acting directly in the mPFC. Bars represent the mean ± standard error of the mean (SEM); *p < 0.05 relative to chance and the vehicle group (*n* = 10–13/group).

To determine whether E_2_ can act directly in the mPFC to enhance memory consolidation, young female mice were ovariectomized and implanted with bilateral guide cannulae aimed at the prelimbic region of the mPFC. One week later, mice were trained in the OR or OP tasks, and then immediately received bilateral mPFC infusion of vehicle or 5 µg/hemisphere E_2_ (*n* = 10–13/group). OR memory was tested 48 h after training. Mice infused with E_2_ (*t*_(12)_ = 3.4, *p* = 0.005), but not vehicle (*t*_(11)_ = 0.18, *p* = 0.87), spent significantly more time than chance with the novel object during testing (Fig. 1*A*,*C*), indicating that only E_2_-infused mice displayed intact memory for the familiar training object. E_2_-infused mice also spent significantly more time with the novel object than vehicle-infused mice (*t*_(23)_ = 2.294, *p* = 0.03; Fig. 1*C*), suggesting that E_2_ in the mPFC enhances OR memory consolidation. OP memory was tested 24 h after training. As in OR, E_2_-treated mice spent significantly more time than chance with the moved object (*t*_(10)_ = 5.06, *p* = 0.001), whereas vehicle-treated mice did not (*t*_(9)_ = 0.18, *p* = 0.9; Fig. 1*B*,*D*). Moreover, E_2_-infused mice spent significantly more time with the moved object than vehicle-infused mice (*t*_(19)_ = 2.5, *p* = 0.02; Fig. 1*D*), indicating that E_2_ in the mPFC also enhances spatial memory consolidation. Together, these data demonstrate that direct infusion of E_2_ in the mPFC enhances OR and spatial memory consolidation in ovariectomized female mice.

### mPFC E_2_ infusion increases mPFC, but not CA1, spine density 2 h after infusion

**Figure 2.**
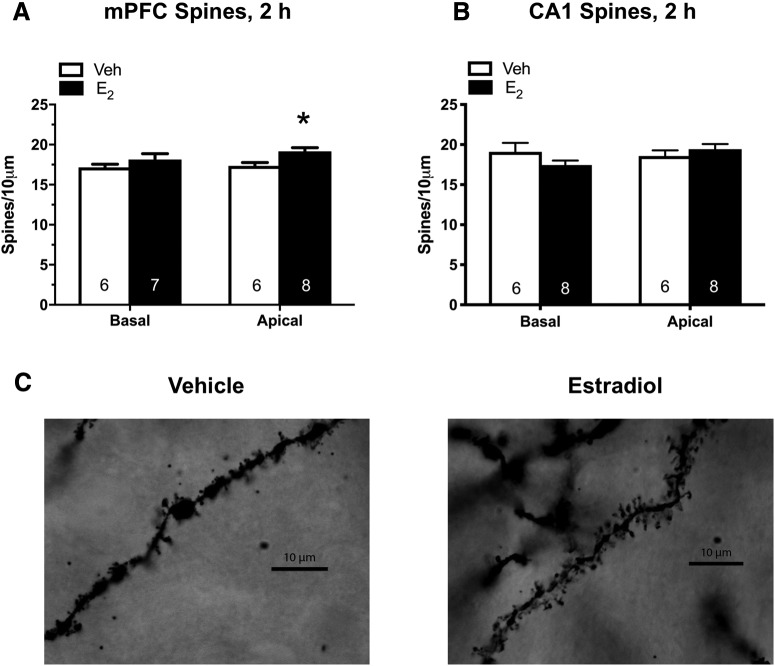
mPFC E_2_ infusion increases apical spine density in the mPFC 2 h later. Relative to vehicle (Veh), apical but not basal, spine density was significantly increased in the mPFC 2 h after mPFC infusion of 5-µg E_2_ per hemisphere (***A***). mPFC infusion did not alter apical or basal spine density in CA1 2 h after mPFC infusion of E_2_ (***B***). Bars represent the mean ± SEM; *p < 0.05 relative to the vehicle group (*n* = 6–8/group). ***C***, Representative photomicrographs of Golgi-impregnated secondary apical dendrites from pyramidal cells in Layer II/III of the mPFC. Under oil 100×.

Two weeks after the completion of behavioral testing, mice received mPFC infusions of vehicle or E_2_ as above, and whole brains were collected 2 h later for Golgi staining and dendritic spine density analyses of the mPFC and CA1 of the DH. We focused our analyses on Layers II/III of the mPFC and the CA1 subfield of the DH, as previous research examining the impact of systemic E_2_ injection, E_2_ fluctuations across the estrous cycle, and ER agonists on spine density have identified these regions as being particularly sensitive to E_2_ (Woolley et al., 1990; Woolley and McEwen, 1992; Phan et al., 2011; Frankfurt and Luine, 2015; Tuscher et al., 2016a). E_2_ significantly increased mPFC apical (*t*_(12)_ = 2.89, *p* = 0.014), but not basal (*t*_(11)_ = 1.11, *p* = 0.28), spine density relative to vehicle 2 h after mPFC infusion (Fig. 2*A*,*C*). No significant differences were observed in CA1 apical (*t*_(12)_ = 0.88, *p* = 0.4) or basal (*t*_(12)_ = 0.02, *p* = 0.98) spine density between vehicle and E_2_ mice after mPFC E_2_ infusion (Fig. 2*B*). These findings demonstrate that E_2_ can increase apical spine density in the mPFC within 2 h of infusion, but does not impact CA1 spine density at this time point.

### Chemogenetic suppression of the mPFC immediately after training prevents E_2_ in the DH from enhancing memory consolidation

**Figure 3.**
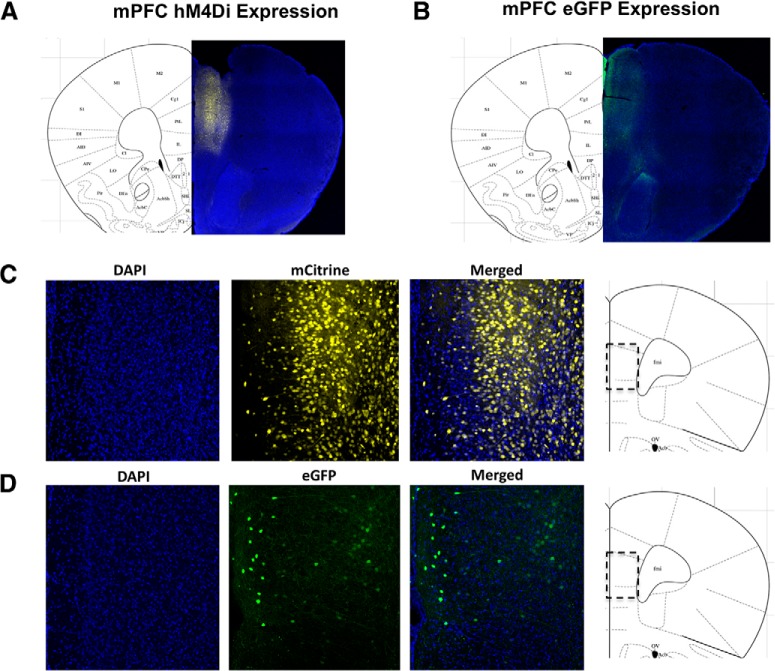
hM4Di and eGFP viral expression in the mPFC is present three weeks after injection. Coronal sections (40 µm) of CaMKII*α*-hM4Di-mCitrine DREADD (***A***, ***C***) or CaMKII*α*-eGFP control virus (***B***, ***D***) in the mPFC of female mice three weeks after injection confirms virus expression throughout the mPFC. Blue puncta: DAPI, a marker of cell nuclei; yellow: mCitrine-tagged DREADD virus; green: eGFP-tagged control virus. Merged images provide additional spatial context for DREADD or control virus expression within the region of interest.

DH infusion of E_2_ increases dendritic spine density in the mPFC (Tuscher et al., 2016a), raising the possibility that the DH and mPFC might interact to mediate the memory-enhancing effects of DH E_2_ infusion. To determine whether the mPFC is necessary for the memory-enhancing effects of DH E_2_ infusion, we suppressed the activity of mPFC excitatory neurons using inhibitory hM4Di DREADDs. Ovariectomized mice were implanted with bilateral DH guide cannulae, and bilaterally injected with saline (sham condition), or an AAV8 viral vector containing either eGFP or the hM4Di DREADD into the mPFC. This viral construct is driven by the CaMKIIα promoter, which allows for selective expression predominantly in excitatory neurons, and suppression of excitatory neural activity near the site of injection when bound by its ligand CNO (Armbruster et al., 2007; Zhu et al., 2014). The control construct AAV8-CaMKIIα-eGFP, which is driven by the same promoter as that used for the DREADD virus but lacks the hM4Di gene (Zhu et al., 2014), was used to control for nonspecific virus effects. A subset of mice (*n* = 3/group) were perfused three weeks after surgery to verify that DREADD and eGFP control viruses were expressed in the mPFC at the initiation of behavioral training (Fig. 3). Expression of the eGFP control (Fig. 3*B*,*D*) and mCitrine-labeled hM4Di (Fig. 3*A*,*C*) viruses was localized to the mPFC, and was detected throughout the dorsal-ventral extent of the mPFC injection site, starting in the infralimbic region and extending throughout the prelimbic region and anterior cingulate in both eGFP control and DREADD-expressing mice, respectively.

**Figure 4.**
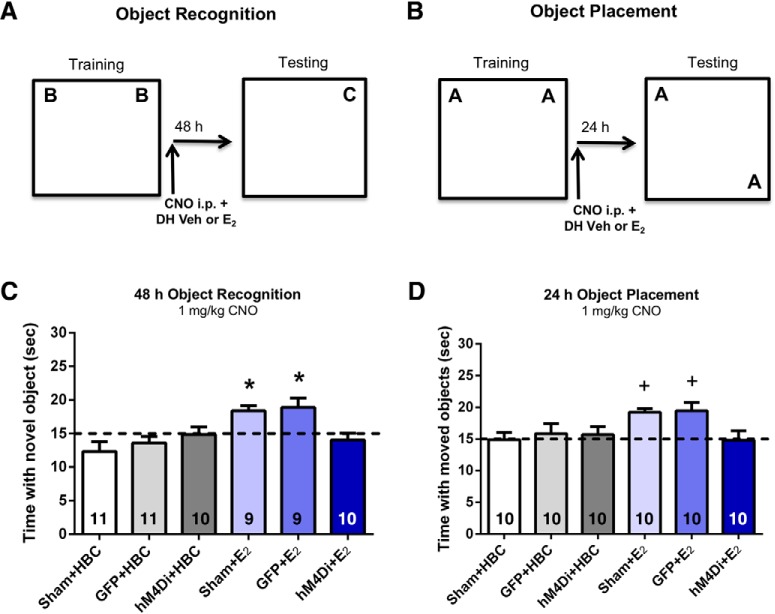
Chemogenetic suppression of the mPFC immediately after training prevents the memory enhancement induced by DH E_2_ infusion. Sham, eGFP, or hM4Di control groups receiving CNO+Veh did not spend significantly more time than chance (15 s) with the novel object when tested 48 h after OR training (***A***, ***C***) or with the displaced object (***B***, ***D***) 24 h after training. In contrast, sham or eGFP mice receiving CNO+E_2_ immediately after training did spend significantly more time than chance with the moved and novel objects, displaying intact OP and OR memory. However, hM4Di mice treated with CNO+E_2_ immediately after training did not demonstrate intact memory, suggesting that DREADD-mediated suppression of the mPFC blocks the beneficial mnemonic effects of DH-infused E_2_. Bars represent the mean ± SEM; *p < 0.05 relative to chance, all Veh-infused groups, and the hM4Di-E_2_ group (*n* = 9–11/group); +*p* < 0.05 relative to chance and the Sham+Veh-infused and hM4Di-E_2_ groups (*n* = 9–11/group).

After establishing viral expression, we next examined whether mPFC activity is necessary for the memory-enhancing effects of E_2_ infused into the DH. To address this question, we used a 1 mg/kg dose of CNO that does not impair memory consolidation on its own in ovariectomized female mice expressing mPFC-hM4Di DREADDs (Tuscher et al., 2018). Ovariectomized mice implanted with bilateral DH guide cannulae and injected with saline, eGFP, or hM4Di underwent behavioral training (*n* = 9–11/group). Immediately after training, mice were injected with 1-mg/kg CNO and then received DH infusion of vehicle or 5 µg/hemisphere E_2_. DREADD-mediated suppression of the mPFC immediately after training prevented E_2_-mediated enhancement of OR memory. Specifically, Sham, eGFP, or hM4Di control groups receiving CNO+Veh did not spend significantly more time than chance with the novel object when tested 48 h after OR training (sham: *t*_(10)_ = 1.85, *p* = 0.09; eGFP: *t*_(10)_ = 1.42, *p* = 0.19; hM4Di: *t*_(9)_ = 0.14, *p* = 0.89; Fig. 4*A*,*C*), suggesting a lack of intact OR memory in all groups infused with vehicle into the DH. In contrast, sham and eGFP mice receiving CNO+E_2_ remembered the familiar training object 48 h later (sham: *t*_(8)_ = 4.37, *p* = 0.002; eGFP: *t*_(8)_ = 2.78, *p* = 0.02; Fig. 4*C*), indicating that sham surgery and the eGFP construct did not prevent DH-infused E_2_ from enhancing OR memory consolidation. Importantly, the hM4Di group receiving CNO+E_2_ did not spend more time than chance with the novel object (hM4Di: *t*_(9)_ = 0.94, *p* = 0.37; Fig. 4*C*) during testing, demonstrating that excitatory neural activity in the mPFC is necessary for DH-infused E_2_ to enhance OR memory consolidation. These findings were further supported by one-way ANOVA, which demonstrated a significant main effect of treatment among the six groups (*F*_(5,54)_ = 5.14, *p* = 0.001). *Post hoc* tests revealed that sham and eGFP groups receiving DH-E_2_ infusions spent significantly more time with the novel object than E_2_-treated hM4Di mice or any group infused with vehicle (*p*s < 0.05; Fig. 4*C*).

To determine whether mPFC-DH interactions are also necessary for spatial memory consolidation, the same mice were tested in the OP task. OP training was conducted just as OR, although the order of testing varied among mice as described in Materials and Methods. Immediately after training, mice were injected with 1-mg/kg CNO, followed by bilateral DH infusion of vehicle or 5 µg/hemisphere E_2_. As with OR, we found that DREADD-mediated suppression of excitatory neural activity in the mPFC immediately after training blocked E_2_-mediated enhancement of OP memory consolidation. Sham, eGFP, and hM4Di control groups receiving CNO+Veh did not spend significantly more time than chance with the moved object when tested 24 h after OP training (sham: *t*_(9)_ = 0.07, *p* = 0.94; eGFP: *t*_(9)_ = 0.56, *p* = 0.591; hM4Di: *t*_(9)_ = 0.56, *p* = 0.59; Fig. 4*B*,*D*), suggesting that spatial memory was not intact in all vehicle-infused groups. In contrast, sham and eGFP mice receiving CNO+E_2_ did spend significantly more time than chance with the moved object 24 h after training (sham: *t*_(9)_ = 7.50, *p* < 0.0001; eGFP: *t*_(9)_ = 3.34, *p* = 0.01; Fig. 4*D*), indicating that E_2_ enhanced spatial memory consolidation in both groups. However, as in OR, DH E_2_-mediated memory enhancement was suppressed in the hM4Di group receiving CNO+E_2_, as this group did not spend more time than chance with the moved object (hM4Di: *t*_(9)_ = 0.13, *p* = 0.90; Fig. 4*D*) during testing. Similar to OR, this pattern of findings was further supported by a significant main effect of treatment (*F*_(5,54)_ = 2.79, *p* = 0.03; Fig. 4*D*) and *post hoc* tests revealed that sham or eGFP mice receiving DH E_2_ infusion spent significantly more time with the moved object than hM4Di mice infused with E_2_, or sham mice infused with vehicle (*p*s < 0.05; Fig. 4*D*). Together, these data suggest that mPFC neural activity is necessary for E_2_ infused into the DH to enhance spatial and OR memory consolidation in ovariectomized mice, which illustrates the importance of systems-level coordination for the memory-enhancing effects of E_2_ in females.

## Discussion

The hippocampus has been the focal point of much neuroendocrinology research examining how E_2_ mediates memory formation and neuroplasticity. However, E_2_ also impacts other brain regions important for regulating cognitive function in females, including the mPFC, amygdala, striatum, and perirhinal cortex (Zurkovsky et al., 2007; Gervais et al., 2013; Maeng et al., 2017). Here, we found that mPFC infusion of E_2_ significantly increased mPFC apical spine density and enhanced both OR and spatial memory consolidation. These data are the first to demonstrate that E_2_ can act directly within the mPFC to facilitate memory consolidation and spinogenesis in female mice. Our findings are consistent with previous work demonstrating that DH E_2_ infusion enhances memory consolidation and increases dendritic spine density in the DH and mPFC within 2 h (Fernandez et al., 2008; Fortress et al., 2013; Tuscher et al., 2016a). Moreover, the extent to which E_2_ infusion into the mPFC influences memory consolidation and spine density is similar to that observed after DH E_2_ infusion in this study and previous work (Fortress et al., 2013; Tuscher et al., 2015, 2016a). Our data also align with previous reports of increased mPFC spine density and memory enhancement in the radial arm maze, Y maze, OR, and OP tasks after systemic E_2_ injection (Inagaki et al., 2012; Luine, 2016). However, the memory-enhancing effects of E_2_ could not be directly attributed to actions within the mPFC in these studies, due to the systemic nature of delivery. Here, we found that direct mPFC E_2_ infusion significantly enhanced memory and increased mPFC apical spine density, suggesting that the mPFC plays a critical role in E_2_-mediated memory enhancement. Although DH infusion of E_2_ significantly increased basal mPFC spine density 2 h after infusion in a previous study (Tuscher et al., 2016a), mPFC infusion of E_2_ in the present study did not reciprocally affect CA1 spine density 2 h later. These data may suggest that DH projections exert greater control over the mPFC than the mPFC exerts in return, or that mPFC input back to the DH is indirect or occurs over a longer timeframe. However, additional time points would need to be evaluated before concluding that mPFC input has no effect on DH spine density.

The specific mechanisms through which E_2_ regulates memory and spinogenesis in the mPFC are currently unclear, as the pharmacological and chemogenetic approaches used in the present study do not allow for clear disambiguation between classical genomic and rapid non-classical actions of E_2_. However, given the necessity of ERK activation for E_2_-mediated spine changes in cortical neurons (Srivastava et al., 2008), and the requirement of ERK and mTOR activation in the DH for E_2_-mediated memory enhancement and spinogenesis (Fernandez et al., 2008; Fortress et al., 2013; Tuscher et al., 2016a), we predict that rapid non-classical mechanisms are also likely critical for the mnemonic effects and spine changes observed after mPFC E_2_ infusion. The observation that significant increases in mPFC spine density occur as early as 2 h after infusion (Fig. 2*A*) also supports the notion that these effects are mediated via the rapid non-classical mode of E_2_ action, although this remains to be tested directly. Future studies involving the infusion of cell-signaling inhibitors directly into the mPFC in the presence of E_2_ will be necessary to more definitively discriminate between classical and non-classical mechanisms. The specific ERs mediating these effects also remain to be elucidated. ERα, ERβ, and G-protein-coupled ER (GPER) are all expressed throughout the mPFC (Almey et al., 2014), and recent work suggests that rapid E_2_-induced hippocampal spinogenesis is mediated in part by ERα and GPER (Phan et al., 2015). Therefore, these receptors are prime candidates for further examination.

In addition to the spine density changes observed in the mPFC after E_2_ infusion alone, there could be “additive” or “synergistic” effects of E_2_ and learning on spine density, as experience-induced changes have been observed in the mPFC (Kolb et al., 2008; Kolb and Gibb, 2015; Ouhaz et al., 2017), although not specifically after training in OR or OP. In the present study, we waited until two weeks after the completion of behavioral training to infuse our respective treatments, and infusions of vehicle or E_2_ occurred in the absence of training immediately before tissue collection. Therefore, even if learning-induced spine changes occur after object training, and such theoretical changes persisted for two weeks after the completion of these tasks, this variable would be consistent across both vehicle and E_2_ treated groups. Therefore, it is unlikely that any additive effects between E_2_ and learning would be observed in this dataset, as any potential experience-induced increases would likely have occurred in both treatment groups. Nonetheless, the possibility remains of additive or synergistic effects between learning and E_2_ on spine density, which could be investigated in future studies.

Our findings also demonstrate for the first time that mPFC neural activity is essential for the memory-enhancing effects of DH E_2_ infusion, and illustrate a novel systems-level relationship between these brain regions in mediating the mnemonic effects of E_2_. This notion is supported by our data demonstrating that suppressing neural activity in the mPFC with the inhibitory hM4Di DREADD immediately after training prevented DH-infused E_2_ from enhancing memory consolidation. The beneficial effects of E_2_ on memory formation appear specific to the consolidation period, as previous research has shown that delayed systemic or DH administration of E_2_ (i.e., 1.5–3 h after training) does not enhance memory consolidation in the OR and OP tasks (Walf et al., 2006; Frye et al., 2007; Fernandez et al., 2008), suggesting E_2_ action during the first few hours after training is essential for memory consolidation. Thus, E_2_’s effects in the DH, and subsequent downstream interactions with other brain regions such as the mPFC after DH E_2_ infusion, appear to facilitate memory specifically during the consolidation period, rather than during recall. This notion is consistent with previous work demonstrating that performance in a fear extinction task requiring the mPFC is facilitated when systemic E_2_ is administrated immediately, but not 4 h, after fear extinction training (Zeidan et al., 2011). Thus, although effects of post-training E_2_ on recall cannot be entirely discounted in the present study, previous data make this explanation highly unlikely.

To ensure that our observed effect on memory consolidation reflected DREADD-mediated suppression of the memory-enhancing effects of E_2_, rather than DREADD-mediated memory impairment in general, we used a dose of CNO that does not impair memory on its own in combination with mPFC-hM4Di DREADD expression (Tuscher et al., 2018), which was essential given recent findings showing that the CNO metabolite clozapine affects locomotion in rats at high doses (Gomez et al., 2017). The fact that chemogenetic disruption of the mPFC prevented the beneficial mnemonic effects of DH-infused E_2_ suggests that these regions coordinate during object memory consolidation, and lend further behavioral relevance to the E_2_-mediated spine changes recently observed in the mPFC after DH E_2_ infusion (Tuscher et al., 2016a). Moreover, this work suggests a new interpretation of previous reports showing that DH infusion of E_2_ enhances OR and spatial memory consolidation (Fernandez et al., 2008; Boulware et al., 2013; Fortress et al., 2013), as the present findings demonstrate the essential involvement not only of the DH, but also of the mPFC. Examining these putative circuit-level interactions may hold the key to understanding how E_2_ regulates memory consolidation.

Many questions remain regarding how the hippocampus and mPFC might interact to facilitate memory consolidation. Tract tracing studies support the existence of several potential routes of communication between the hippocampus and mPFC, including direct unilateral projections from dorsal CA1 and subiculum to the mPFC (Hoover and Vertes, 2007), unilateral projections between the ventral hippocampus and subiculum to the mPFC (Ferino et al., 1987; Jay et al., 1989; Jay and Witter, 1991; Cenquizca and Swanson, 2007), and indirect reciprocal connections routed through the nucleus reuniens of the thalamus or lateral entorhinal cortex (Burwell and Amaral, 1998; Hoover and Vertes, 2007; Vertes et al., 2007). Evidence for the functional relevance of these connections is supported by electrophysiological studies demonstrating that stimulation in the ventral CA1/subiculum of anesthetized rats results in stable long-term potentiation (LTP) in prefrontal neurons (Laroche et al., 1990; Jay et al., 1992). LTP between hippocampal and prefrontal synapses leads to a persistent increase in synaptic strength in awake behaving rats (Jay et al., 1996), suggesting the existence of direct excitatory input from the hippocampus to the mPFC. Other evidence suggests that temporall*y*-coordinated neuronal activity occurs between the hippocampus and mPFC during periods of wakefulness and sleep in rodents, and that this synchronous activity is essential for memory consolidation (Jones and Wilson, 2005; Hyman et al., 2010; Sigurdsson et al., 2010; Schwindel and McNaughton, 2011). As such, it follows that chemogenetic suppression of the mPFC in the present study may have disrupted functional connectivity between the DH and mPFC, thereby disrupting systems-level processes through which E_2_ facilitates memory consolidation in female mice.

Although the mechanisms through which these circuit-level changes occur are currently unclear, DH-infused E_2_ may promote the transcription of activity-dependent genes in projection regions such as the mPFC, and DREADD-mediated mPFC suppression might prevent these alterations from occurring. The idea that neuronal activity in the DH can alter gene expression in projection regions essential for memory formation is supported by at least two recent studies. In one, disruption of DH input to the mPFC during fear conditioning prevented later reactivation of putative mPFC engram cells and training-induced increases in mPFC spine density (Kitamura et al., 2017). In another study, injection of Arc antisense oligonucleotides into the DH blocked fear reactivation-induced increases in neural activity markers (e.g., Arc, pCREB, and pCofilin) in both the DH and mPFC, and prevented reactivation-induced enhancement of fear memory expression (Ye et al., 2017). Further, DREADD-mediated inhibition of DH projection terminals in the mPFC before reactivation sessions also prevented reactivation-induced increases in fear memory expression and memory-associated proteins in the mPFC (Ye et al., 2017). Both studies indicate that inhibiting either neural activity or translation of neural activity markers within the DH alters cellular activity and spine density in the mPFC, and that disruption of these processes during consolidation impairs long-term memory. These findings are consistent with previous reports that ERK or mTOR inhibition in the DH are necessary for DH-infused E_2_ to enhance memory consolidation and increase mPFC spinogenesis in ovariectomized mice (Fernandez et al., 2008; Fortress et al., 2013; Tuscher et al., 2016a). Together, these findings provide evidence that molecular processes in the DH (e.g., cell-signaling activation, protein translation) influence the mPFC. The present findings expand on this work by demonstrating that disrupting mPFC-DH interactions prevents DH E_2_ from facilitating memory consolidation. These data collectively support a model in which DH infusion of E_2_ increases excitatory input to the mPFC, which then drives activity-dependent changes in gene expression and/or protein translation essential for spinogenesis and memory consolidation. Chemogenetic suppression of neural activity in the mPFC may blunt the E_2_-induced increase in excitatory input, thereby preventing potential synaptic connections between the DH and mPFC from being strengthened after E_2_ infusion, which in turn could prevent estrogenic enhancement of memory consolidation.

In sum, converging lines of research support a role for estrogenic modulation of hippocampal physiology, neuroplasticity, and memory consolidation. However, ERs are expressed in numerous brain regions, including the mPFC, that act in concert with the hippocampus to modulate cognitive function. Here, we provide evidence that E_2_ within the mPFC can also regulate mPFC spine density and facilitate memory consolidation in female mice, and that E_2_-mediated enhancement of memory requires communication between the DH and mPFC. Such work provides key insights into how the hippocampus coordinates with other brain regions to facilitate the estrogenic regulation of memory. Further, given that coordination between the hippocampus and PFC is compromised in several psychiatric disorders and neurodegenerative diseases for which females are at greater risk (i.e., depression, post-traumatic stress disorder (PTSD), Alzheimer's disease (AD); Tolin and Foa, 2006; Dye et al., 2012; Godsil et al., 2013; Albert et al., 2015; Sampath et al., 2017), this work also provides a critical foundation for dissecting the circuit-level basis of certain mental health disorders, and may aid in the development of systems-level therapeutic strategies.

## Synthesis

Reviewing Editor: Rae Silver, Columbia University Decisions are customarily a result of the Reviewing Editor and the peer reviewers coming together and discussing their recommendations until a consensus is reached. When revisions are invited, a fact-based synthesis statement explaining their decision and outlining what is needed to prepare a revision will be listed below. The following reviewer(s) agreed to reveal their identity: Jessica Mong, Elena Choleris.

This study investigates the role of the medial prefrontal cortex (mPFC) in estradiol (E2) mediated effects on memory. Previous work suggested that E2 facilitates the coordination between the dorsal hippocampus (DH) and mPFC to enhance memory functions. However, little is known about how E2 is acting in the DH-mPFC circuit. This is a novel description of the interplay of two brain regions, the dorsal hippocampus and the medial prefrontal cortex in the mediation of estrogen enhancement of the consolidation of long term memory. More specifically, the authors using a mouse model of object recognition/object placement test whether (1) E2 acts directly in the mPRC to regulate memory function and (2) the extent at which the DH-mPFC circuit is necessary for E2 enhanced memory. To test these aims the authors first infused E2 directly into the mPFC of OVX mice. Direct infusion of E2 into the mPFC increased apical spine density and enhanced object recognition and spatial memory consolidation and inactivation of the mPFC blocked DH-infused E2 from enhancing object recognition and spatial memory consolidation. These findings provide evidence that the mPFC is necessary in E2-regulation of memory functions both directly and through the interactions with the DH. Overall, this is a well-written and straight forward study that suggests a novel direct estrogenic action in mPFC which is necessary for enhanced memory. However, there are a few minor concerns and comments. Finally, the authors can be commended for using (and emphasizing the importance of using) doses of CNO that per se do not affect memory. That is the only correct way of showing a true interplay with estrogens. Unfortunately, this approach is not used in all published research.

A number of observations and questions have been raised by the reviewers, each of which should can be addressed in a revision.

1. In the abstract and the significance statements the DP -> mPFC link is not developed, other than hypothesizing “other brain regions like the mPFC”. A more hypothesis driven reason for looking at the mPFC would read better (and better reflect the main text of the manuscript)

2. It would be helpful to know if direct infusion of E2 into the mPFC results in an equivalent level of enhancement when compared to infusion into the DH or systemic administration.

3. Is it known whether using excitatory DREADDS in the mPFC can mimic the E2 effect? This would further support a direct action of the mPFC on memory function. Also, is a functioning DH necessary for the direct mPFC E2 effect on memory? Adding any such knowledge to the discussion would be useful in thinking about E2 role in the mPFC.

4. On some technical notes: It does not appear that the GFP control virus had similar levels of expression. The mCitrine is more robust. Also, in the photomicrographs (in c and d) it is not clear what the merged images are representing.

5. While the authors cite an article in press demonstrating that CNO alone does not have any effect on memory, it is not entirely clear that this control was run in the presence of E2.

6. Photomicrographs of the golgi- impregnated dendrites and representative differences in spine densities should be included.

7. Line 73. “both types of memory” is unclear here. Please specify/repeat, OR OP.

8. The same mice were used for learning and golgi staining. Could learning per se induce dendritic spines and could this effect interact with that of E2?

9. A justification for the choice of the specific DH subregion (CA1) and PFC layers (II/III) would be helpful for the reader to contextualize this work.

10. A description of the DREDD expression would be useful. How specific was it for the targeted area? Was any staining found in nearby regions? Also, what was the efficacy of transfection (No of neurons expressing GFP)? How about cell type specificity? Could this be assessed?

11. Discussion. Can the authors definitively conclude their effects are on memory consolidation vs long-term memory retention/recall? Their E2 treatment was administered immediately after learning. Would it be effective even if it was administered after some times, after the closure of the so-called consolidation window?

12. Discussion. There is no mention as to whether the observed effects are mediated by rapid vs classical effects of estrogens. The molecular mechanisms discussed imply the authors are thinking in terms of rapid effects, however the discussion does not directly address this question.
